# Physiotherapeutic Strategies and Their Current Evidence for Canine Osteoarthritis

**DOI:** 10.3390/vetsci10010002

**Published:** 2022-12-21

**Authors:** Monika Anna Mille, Jamie McClement, Susanne Lauer

**Affiliations:** 1 Centre for Clinical Veterinary Medicine, Ludwig Maximilians University, 80539 Munich, Germany; 2 Abington Park Veterinary Referrals, Northampton NN3 7RR, UK

**Keywords:** osteoarthritis, physiotherapy, dog

## Abstract

**Simple Summary:**

Osteoarthritis (OA) is a chronic progressive disease, which frequently requires lifelong therapy in dogs. In addition to pain management with drugs, exercise modification and physical therapy are important measures to alleviate pain and to improve patient mobility and quality of life in osteoarthritic dogs. The main goals of physiotherapy for osteoarthritic dogs are pain reduction, improvement of muscle strength and preservation of joint function. For this purpose, the patient’s life style and environment are optimally adapted to facilitate daily life in osteoarthritic dogs. Owners learn to optimize daily exercise and to perform simple home exercises. Additionally, physiotherapists regularly adapt the PT-program according to patient progress and complement the home program with “PT-expert” exercises and physiotherapeutic modalities (for example: shock wave or laser therapy) to further improve the patient’s overall quality of life and function. The authors propose a simple systematic PT approach for canine OA, prioritizing measures according to simplicity, cost effectiveness and practicality in the form of a “PT pyramid”. The levels of the pyramid are in ascending order: environmental modification, exercise plan, OA-specific home exercises, and treatment by a veterinary physical therapist. Additionally, efficacy of physiotherapeutic measures is reviewed for canine osteoarthritis.

**Abstract:**

Osteoarthritis (OA) is a common and debilitating condition in domestic dogs. Alongside pharmaceutical interventions and weight loss, exercise and physiotherapy (PT) are important measures in maintaining patient mobility and quality of life. Physiotherapy for OA aims to reduce pain, optimize muscular function and maintain joint function. Physiotherapeutic plans typically include PT exercises combined with therapeutic modalities, lifestyle and environmental modifications to improve the patient’s overall quality of life and function. Information on therapeutic clinical efficacy of physiotherapeutic measures for canine OA is still very limited. Thus, physiotherapeutic strategies are still primarily based on evidence extrapolated from human protocols tailored to people with OA. The authors propose a simple systematic PT approach for canine OA, prioritizing measures according to simplicity, cost effectiveness and practicality. This guide (the “Physiotherapy Pyramid”) aims to provide a clear stratified approach to simplify decision making and planning for owners, veterinarians and veterinary physiotherapists, leading to more straightforward design and implementation of treatment plans. Measures are implemented starting at the base of the pyramid, subsequently progressing to the top, allowing effective and practical interventions to be prioritized. The levels of the pyramid are in ascending order: environmental modification, exercise plan, OA-specific home exercises and treatment by a veterinary physiotherapist.

## 1. Introduction

Osteoarthritis (OA) is a degenerative joint disease with a high prevalence in dogs [1,2]. Due to its chronic progressive and potentially disabling nature, OA may impact quality of life of dogs and their owners negatively [3,4,5]. Current therapeutic strategies for canine osteoarthritis are multifaceted, including pharmaceutical analgesic interventions, nutraceutical approaches, intra-articular and disease-modifying treatments, regenerative medicine, exercise modification and physiotherapy [6]. The interest of dog owners in physiotherapy as an adjunctive or alternate physiotherapeutic treatment option has substantially increased throughout the last two decades [3,7,8]. Particularly in geriatric osteoarthritic dogs, that have become non-responsive to “standard” therapy (in general refers to medical pain management, weight loss and controlled low impact exercise), physiotherapy is often considered a last resort with the goal of pain relief and improved quality of life. Consultations and decision making upon optimal physiotherapeutic measures for dogs with OA can be challenging for both owners and veterinarians, considering average duration of consultations in small animal practice and the multitude of available options [3,9,10]. Multimodal OA therapy can be very costly, time consuming and difficult to integrate into daily life [9]. Moreover, currently, evidence-based information on physiotherapeutic interventions for dogs with osteoarthritis is mostly still limited to empirical guidelines from textbooks [11,12,13] and derived from human and experimental studies. Thus, considering the current situation, the authors propose a simple systematic PT approach for canine OA, prioritizing measures according to simplicity, cost effectiveness and practicability. This guideline (the “Physiotherapy Pyramid”) aims to provide a clear stratified approach to simplify decision making and planning for owners, veterinarians and veterinary physiotherapists, leading to more straightforward design and implementation of treatment plans.

In this review, the authors discuss the different levels of the pyramid (environmental modification, exercise plan, OA-specific home exercises and treatment by a veterinary physiotherapist) taking into account the current literature. To this end, evidence on the topics addressed below was collected using the databases Pubmed, Google scholar and PEDro without setting limits for date or type of publication. The results were taken into account weighted by the strength of evidence ranging from systematic reviews, RCTs, experimental studies to observational studies, but without considering mere individual opinions or subjective experience.

## 2. Role of Physiotherapy in Management of Osteoarthritis

Osteoarthritis triggers progressive loss of joint cartilage, synovial inflammation, formation of osteophytes, and subchondral bone remodeling subsequent to complex multifactorial catabolic processes [14,15]. Structural disintegrity of the osteoarthritic cartilage ultimately causes joint stiffness, pain and limits joint range of motion throughout the course of disease [15]. Continued movement and physiologic mechanical loading of joints has been considered vital for joint health and cartilage repair, as expression of proinflammatory genes and inflammatory signalling cascades is inhibited [16]. Decreased patient activity subsequent to OA pain typically results in impaired periarticular muscle function, thus triggering a vicious cycle [17]. Effective physiotherapeutic interventions counteract this vicious cycle, enhancing muscle strength, neuromuscular control, range of motion, joint stability and fitness in OA patients [18]. Based on international guidelines, physiotherapy is overall recommended for management of OA in people in primary care facilities [19,20]. Physical exercise has been shown to improve mobility and pain scores in people with OA [21]. Structured land-based exercise programs are currently considered core treatments for knees, hips and also polyarticular OA in people [22]. Based on a recent systematic review, both strengthening and aerobic exercise can reduce pain and improve function and health status in people with knee and hip OA [23]. Dependent upon disease stage and the individual patient profile, further physiotherapeutic interventions for example aquatic therapy are considered efficacious [22]. Significant clinical benefits of manual therapy or transcutaneous electrical nerve stimulation could not be determined for hip and knee osteoarthritis in humans [24]. Evidence does not support one exercise modality over another, but supports the role of physical exercise in general [18].

Physical medicine and rehabilitation physicists play a central role in human OA management, as arthritis education is considered essential for successful OA therapy [25]. Both home and group exercise are considered efficacious in people with hip and knee OA [23]. Based on a recent systematic review, cost efficacy has been shown for exercise interventions with or without education for human OA therapy [26]. There is evidence supporting individualized programs for the patient, and although the value of therapy from qualified therapists improves outcomes, they emphasize the value of regular home exercise and the importance of adherence to recommended programmes [18,23,27].

Veterinary physiotherapy is a very young health specialty. Physiotherapeutic recommendations for canine OA are frequently extrapolated from clinical human OA studies or animal models designed for human OA [28,29,30,31,32]. Many canine PT-related studies focus on non- or postsurgical treatment of orthopaedic diseases such as cranial cruciate ligament disease or hip dysplasia associated with secondary OA changes [7,33,34,35]. However, recently, an increasing number of studies also investigate efficacy of modalities or therapeutic exercises for standalone OA [36,37,38,39,40,41,42,43].

## 3. Physical Therapy Pyramid

The primary role in devising management protocols for osteoarthritic patients usually lies with a first opinion veterinary practitioner, rather than a specialist in rehabilitation. Given the numerous PT modalities available, the relative paucity of literature and the amount of direct advertising of services to owners, this role may often be overwhelming, leading to suboptimal management programs. Practitioners feel unable to give clear advice on the relative priority of different modalities due to cost and effectiveness.

**Figure 1:**
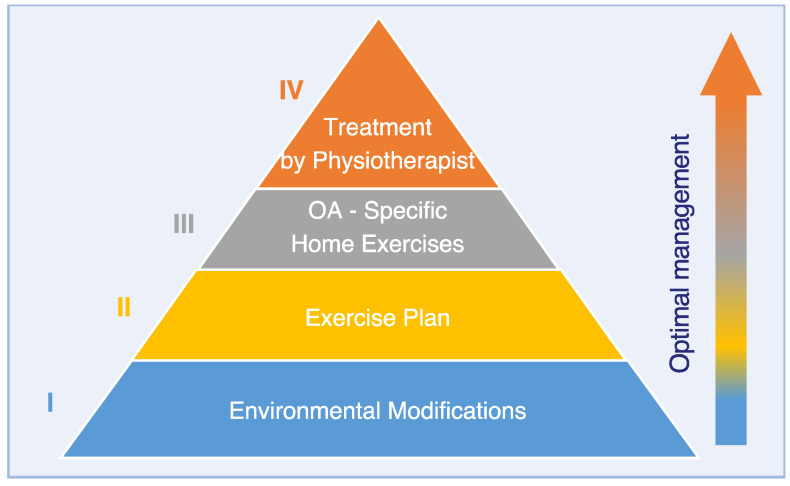
Physiotherapy Pyramid for a systematic approach to canine OA management.

The Physiotherapy Pyramid (Figure 1) has been devised to illustrate a systematic organized approach prioritizing simple measures as a foundation for success in a global approach to OA management. The available modalities have been broadly grouped into layers of the pyramid. Those thought to be more practical, cost-effective and beneficial are at the lower layers should be considered and implemented before those in the layers above—i.e., planning should start at the base of the pyramid, and then subsequently progress towards the top. Patient comfort needs to be taken into consideration throughout each step. Clinicians may then use this structure as a guideline to more confidently recommend modalities to the patient owner in a clear and ordered manner.

### 3.1. Pyramid Level I: Environmental Modification

Evidence for the benefits and methods of environmental modification in canine OA patients is still limited. Thus, recommendations are primarily based on experience and clinical judgement of the veterinary team and patient owner. Recommendations are currently tailored based on specific information on the individual patient and its immediate environment (both home and exercise areas), rather than on evidence-based protocols [44].

Independent locomotion is important for dogs, both from a rehabilitative and behavioural reasons [45]. In severely recumbent patients, or those with minimal ability to rise from rest, soft bedding is clearly recommended to avoid decubital ulceration [46]. Adjuncts such as support slings may be beneficial to aid the owner in helping the patient to rise and ambulate safely [47]. Osteoarthritic patients, due to their reduced muscle mass and reduction in joint proprioceptive receptors, are prone to slipping and often lose confidence in ambulation (or are prone to injury) if the flooring in their home environment does not provide good grip underfoot. Altering slippery floors (either replacing with carpet, or using rubber matting in areas the patient must access) can improve both confidence and ability to move safely within the home [44]. Raising the height of food and water bowls and using ramps to allow access over steps or into cars is also a straightforward adaptation that can improve mobility. Consideration may be given in the home to ensure that the patient is not forced to use steps or stairs to move between food, water, and rest areas. When outside, exercise areas should be chosen with good footing (such as grassland or parks) rather than rough ground, and softer more regular surfaces such as grass are to be preferred over hard and irregular ones (such as gravel, shingle or wet rocks), especially in patients with distal limb OA.

These changes are easily made, but often overlooked. Raising of owners’ awareness by clinicians after questioning or inspection of the home environment may provide significant benefit to the patient complementary to pharmaceutical and PT regimes. As many owners with dogs suffering from osteoarthritis feel lost and not sufficiently supported by their veterinarians due to lack of time during appointments [9], implementing apps helping owners to optimize the home environment may prove useful.

### 3.2. Pyramid Level II: Exercise Regimen/Activity

OA is often interpreted as the result of “wear and tear” [48], leading to the belief that further exercise could worsen the problem. This statement is not true, and OA should be seen as a disease with a more complex, still not completely understood pathogenesis [49]. However, the important role of the cartilage is undisputed. Because its metabolism is only via diffusion through synovial fluid, movement is very important. It has been shown that a moderate running program (10 weeks, 1 h daily on a 15° uphill treadmill, 4 km/h) increases Glycosaminoglycans in the synovial fluid of the stifle in young beagles and thickening of the cartilage [50]. However, to the authors’ knowledge, there is currently a lack of similar studies exploring the effects of walking over ground with differing velocity and duration on cartilage metabolism.

There are currently no studies exploring if dog sports such as agility or fly ball increase the risk of OA. The increased incidence of knee OA in human athletes seems to be the result of injuries, as a prospective study with long-distance runners (with follow-up over 20 years) showed no increased risk of knee OA [51]. A recent systematic umbrella review on the effects of physical activity in human knee and hip OA concluded that there is strong evidence that exercise decreases pain and improves function [52]. In addition, even 45 min per week of moderate-intensity activity is associated with improved function, with effects lasting up to 6 months. A second review assumes that there is no recommendable intensity for general human health benefits: everyone can improve by simply becoming more active, even with relatively minor volume [53]. Comparable studies in veterinary medicine are missing.

In veterinary medicine, the feasibility of an activity monitoring in 72 OA affected dogs was evaluated and rated as a helpful tool [54]. In addition, there was a negative correlation between activity level and body weight. An increased body weight is a well-known risk factor in development of canine OA and weight reduction leads to an improvement of the symptoms (especially lameness) [55,56]. One important fact is the reduction of the load, but additional biochemical factors remain unclear: in obese humans, a pro-inflammatory effect [48] is reported (e.g., pro-inflammatory cytokines), but this could not be confirmed in dogs [57]. Whether hyperleptinemia, which is increased in obese dogs [58], is important for development of OA is unclear.

However, physical activity should be an important factor in OA management and applied with a patient-oriented intensity, regular frequency, and with low impact [59]. Considering the current paucity in the literature, over ground walking is assumed to provide adequate exercise in dogs with osteoarthritis. Often, a specialist is helpful to analyse the current activity level and customize the training to the severity of the OA condition [54]. Excessive activity, which leads to a worsening of the lameness, should be avoided.

### 3.3. Pyramid Level III: OA Specific Home Exercises

#### 3.3.1. Introduction

Home exercises have been shown to be a useful adjunct to programs performed in a clinical setting [21,60,61]. Exercises and treatments provided by physiotherapists and hydrotherapists form an important part of the PT regime in OA treatment but are limited in that they are only performed sporadically when the patient attends the PT centre. Although there are limitations to the level and extent of exercises that can be performed at home, as the majority of dog owners merely have layman’s knowledge of the relevant anatomy, joint motions and restraint, there is the benefit of regularity of these modalities [62,63]. Conscious inclusion of the dog owner into the therapeutic team has the potential to increase compliance [18]. Home exercise programs including especially strengthening, proprioceptive and balancing exercises, as well as massage should be tailored to the dog and take the needs, schedule, preferences and talent of the care taker/s into consideration. It is important that clinicians and therapists ensure that the owner is aware of the need for adequate pain management, and is trained to correctly perform the prescribed exercises without causing discomfort or endangering the patient. Manual therapy performed by physiotherapists and simple active exercises utilizing the patient’s natural repertoire of movements and its body weight should be preferred over those using equipment (such as treadmills, supports and balance boards) to avoid risk of injury.

#### 3.3.2. Strengthening Exercises

Muscle weakness is a key symptom in osteoarthritic people and has been shown to be a predictor of knee OA onset [64,65]. In people with OA, muscle weakness is leading to decreased proprioception and instability in human knees, and there is an increased risk of falling [66]. In people, physical exercise is considered the most frequently recommended non-pharmacologic treatment for OA [19]. Physical exercise not only has the potential to increase muscle strength, but has also been shown to improve proprioception and joint functionality [67]. In particular, strengthening is considered a core treatment for human OA [19], considering the pain reduction, improved physical function and quality of life that can be achieved [68,69]. This is of even greater significance, as muscle strengthening programmes for knee OA have improved joint stability and were able to prevent joint dysfunction based on a six-year cohort study [70]. The clinical efficacy of strengthening exercises in osteoarthritic dogs has not been established yet but are recommended on a routine basis clinically and expected to play a key role in canine OA therapy [71,72]. Strengthening exercises are tailored by the physiotherapist according to the affected osteoarthritic joint and the corresponding muscle weakness. The positive effect of muscle strengthening is expected to become only apparent after weeks to months and needs be maintained with ongoing training [72]. In dogs, resistance training is commonly utilized for strengthening with dogs working against their own weight (for example sit-to-stand exercise) or against additional stressors (for example elastic bands, or carpal weights) [73]. Regular walking is routinely recommended as exercise for osteoarthritic dogs to aid strength, aerobic capability and weight control [18,21,74,75]. Walking on different terrains is an exercise that can be accomplished by most dogs and dog owners.

Depending on the gait phase, many different muscles are activated, both in the trunk and the limbs. Some muscles primarily stabilize (such as the supraspinatus and infraspinatus muscles in the stance phase) and others actively flex or extend [76,77].

The walking exercise can also be adapted to the individual dog through variations such as slight inclines, which has shown to increase the hip joint flexion [78].

There is currently no evidence that the type of strengthening exercise by itself affects outcome in human OA patients significantly [19]. Nevertheless, human guidelines indicate that outcome can be optimized, when strengthening exercises are complemented by other exercises (for example ROM, stretching, functional balance and aerobic exercises). Consistent progression of strengthening is considered a “must” for therapeutic success [69]. These recommendations appear applicable to canine patients too.

#### 3.3.3. Passive Range of Motion (ROM) Techniques

Controlled joint motion is considered vital for joint health [16]. The benefits of joint motion were first demonstrated by R.B. Salter and colleagues in the 1970s in experimental rabbit models comparing effects of continuous joint motion to joint immobilization after cartilage injury [79]. Since then, continuous passive motion (CPM) has been applied in people above all as adjunct therapy after articular surgery with the goal to improve cartilage healing and regeneration and decrease potential for post-traumatic arthritis [16,80,81]. For CPM, range of motion is applied to the joint by an automatic motor-driven device with the goal to allow for early joint movement without the patients needing to use their muscles. Continuous passive motion has been shown to stimulate proteoglycan 4 chondrocyte metabolism in healthy bovine joints [82], but studies specifically investigating the effect of CPM for osteoarthritic joints are scarce [83]. Clinically, CPM combined with thermo- and vibrational therapy has been shown to decrease pain in people with OA [83]. It remains unclear whether this effect is due to CPM itself. Clinically, CPM is currently not performed in dogs. However, a CPM related technique, namely passive range of motion (PROM), is very popular and frequently recommended as home exercise to be performed by owners in osteoarthritic dogs [84]. Passive ROM is the motion of a joint without contraction of a muscle through the available ROM [85]. Currently, there is no direct evidence for efficacy of PROM for OA management and benefits appear to be mainly extrapolated from CPM research. Stretching techniques are also utilized clinically in conjunction with ROM exercises with the goal to elongate shortened periarticular tissues in dogs with restricted joint ROM [85]. Passive stretching of osteoarthritic elbow, carpal and stifle joints performed by owners twice daily for 21 days improved joint range of motion [43]. Longevity of this effect is currently unknown.

Manual joint mobilization techniques require special training for the correct application and are not suitable for laymen. Throughout joint manipulation and stretching, elasticity of the joint capsule and periarticular musculature is improved. Manual joint therapy in the form of joint mobilizations has been shown to reduce pain, stiffness and dysfunction in people with OA, but efficacy in dogs has not been explored yet [86].

Simple passive joint movements [85] performed by physiotherapists or owners are not considered a core element in PT protocols for osteoarthritis, as they are not expected to improve muscle strength. Active exercises are definitely preferred over passive techniques and should be executed whenever possible and tolerated by the patient [19].

#### 3.3.4. Proprioceptive and Balance Exercises

Balance relies on an intact sensorimotor system including proprioceptive acuity and muscle function. A complex visual, vestibular and proprioceptive input is needed for balance control and normal stable gait and functionality [87,88]. Proprioceptive deficits may also trigger onset of human OA and further its progression [89]. Osteoarthritis has been shown to impair proprioceptive perception and postural stability in people [90]. A recent pedobarographic study indicates that OA may also compromise balance and joint proprioception in dogs, as limb centers of pressure pathway characteristics were altered in dogs with osteoarthritic elbows [91]. These balance and postural deficits combined with decreased muscle strength have been associated with an increased risk for injury in people with OA [92]. To the authors’ knowledge, the association of risk for injury and OA in dogs has not been investigated yet.

Balance and proprioceptive training has been shown to effect walking ability, balance, pain, stiffness and functionality positively in people with OA. The effect of balance and proprioceptive training techniques on osteoarthritic dogs has not been investigated yet. However, a recent pilot study training healthy agility dogs on a motorized imoove^®^ balance platform (Allcare Innovations, Bourg-lès-Valence, France) indicates enhanced body control, superior speed during agility trials and improved muscular mass [93] compared to the dogs’ performance prior to the balance training. Further studies are needed to investigate whether and how dogs with OA may benefit from different proprioceptive and balance exercises. As these exercises can be easily implemented by owners at home, the authors propose that proprioceptive and balance exercises should remain components of home exercise protocols for dogs with OA until further evidence arises.

#### 3.3.5. Massage

Massage is a popular therapeutic measure and frequently encountered component within PT programs for osteoarthritic dogs. In people, massage has been shown to increase circumarticular blood circulation, to improve muscular tension and joint flexibility [94]. Additionally, systemic effects such as changes in the vegetative nervous system with reduction of stress and anxiety have been induced by massage [95,96,97]. In people with knee OA, classical massage therapy (effleurage, kneading, rubbing, tapping and vibrating) reduces stiffness and pain, and improves function and ROM [98]. Similar basic canine massage studies have not been performed yet. There is still a lack of studies investigating efficacy of the different massage techniques in dogs. Empirically, massage is frequently used by physiotherapists to habituate and calm osteoarthritic dogs at the beginning of the treatment and for its potential pain-relieving effect [97]. Dog owners are also instructed in basic massage techniques to treat osteoarthritic dogs at home. Studies are needed to explore the effect of basic massage techniques, but also of myofascial release, functional massage therapy and trigger point therapy in osteoarthritic dogs.

#### 3.3.6. Aquatic Exercise

Patient owners may have access to lakes, rivers and oceans during the walks with their dogs. Recently, canine-specific pools have become accessible to dog owners for recreational, therapeutic and conditioning purposes too [99,100]. Based on a recent questionnaire study, 18% of dogs undergoing hydrotherapy in hydrotherapy centres in the United Kingdom are treated for OA [100].

Aquatic therapy has been proposed as an effective therapy form for patients with OA. Buoyancy, hydrostatic pressure, viscosity and turbulence are physical properties that can be exploited therapeutically and allow for high intensity low impact exercise in osteoarthritic dogs, especially if overweight and/or painful [101]. Joint kinematics and stride parameter differ depending on whether dogs are swimming or walking at different water levels on an underwater treadmill [102,103,104]. Recent underwater treadmill studies on healthy dogs reflect that vastus lateralis, biceps femoris and longissimus dorsi muscle activation is affected by water level [105,106].

Current human guidelines recommend aquatic exercise for knee OA, dependent upon comorbidity status, but are not considered efficacious for patients with hip or polyarticular OA [22]. To the authors’ knowledge, clinical efficacy of the different aquatic exercise forms for muscle strengthening has neither been investigated for healthy nor for osteoarthritic dogs. In one recent study, elbow joint ROM increased significantly in dogs with elbow OA after a 20 min swimming session in a pool, when evaluated 10 min after the session [107]. Long-term benefits of aquatic therapeutic interventions with regard to functionality, joint stiffness, comfort and ROM of osteoarthritic dogs have not been investigated yet.

### 3.4. Pyramid Level IV: Treatment by Physiotherapist

#### 3.4.1. Role of a Physiotherapist in Treatment of Osteoarthritis

The physiotherapist’s task as an expert is first to evaluate the patient and its environment, second to develop a treatment plan and third to integrate the plan into the patient’s and its owner’s daily life. The trend goes away from the physiotherapist as a kind of remedy applied to the patient, towards the physiotherapist as a consultant and expert. We should go from working “on” the patient towards working “with” the patient [108] and in veterinary medicine working with the owner too.

#### 3.4.2. Physiotherapeutic Assessment in Canine OA

**Figure 2:**
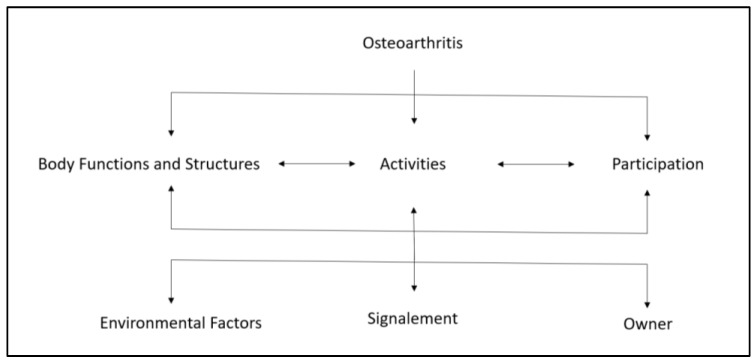
ICF (International Classification of Functioning, Disability and Health) Model modified for OA in veterinary medicine.

The physical effects of OA vary from patient to patient (body functions and structures) and are influenced by comorbidities and by daily activities (walking time, sport activities, owner’s activity). Some limitations have an impact on daily life in which the dog can no longer participate as usual, e.g., jumping into the car. In addition, there are environmental factors, such as only being able to reach the home via stairs, which must be included in therapy planning and goals. Individual factors such as breed and age should also be mentioned. Special attention should be paid to the goals, resources, and compliance of the owner. Although numerous outcome measures are available for canine osteoarthritis [109], there is no suitable diagnostic tool addressing overall function and fitness in veterinary physiotherapy. We therefore propose to adopt and adapt the widely used human diagnostic tool called ICF (“International Classification of Functioning, Disability and Health“), established by the World Health Organization) for the use in canine OA. This assessment tool considers all aspects of functioning [110] in daily life (see Figure 2).

Based on this holistic functional assessment, the physiotherapist develops an individual exercise plan. In human medicine, supervised active treatment (exercises) is currently considered best practice [111] when physiotherapy is initiated in patients with knee and hip osteoarthritis. A minimum of 12 sessions are recommended (two per week) with active treatment progressing in intensity over time and development of a home-exercise program to obtain sufficient clinical benefit [111]. Fewer than 12 sessions are less effective among human knee OA patients [112]. Adjunct treatments such as electrotherapy are only recommended after this initial phase, if the patient’s symptoms do not improve sufficiently.

Other possible techniques, such as taping, functional massage therapy (combination of joint motion and massage), proprioceptive neuromuscular facilitation stretching or manual joint therapy, have to be applied by the physiotherapist and are not suitable for home-exercise programs [18,113,114]. Manual joint therapy requires knowledge about joint anatomy, movements and an experienced practitioner. Manual joint therapy has shown a better outcome regarding pain in human OA than exercises alone [68] and is often successfully used in the treatment of osteoarthritis in people [60,115]. Taping and proprioceptive neuromuscular facilitation stretching is common in human OA physiotherapy, but not yet well established in pets. Despite reported positive experience of individual practitioners, there is still a lack of evidence in veterinary medicine.

Exercises must be performed correctly to be effective in OA patients. Therefore, regardless of the chosen exercise, it has to be prescribed appropriately [116] and as specifically as possible [117]. In veterinary medicine, the correct execution of exercises may be challenging, as concise oral instructions are difficult when working with canine patients. The supervising physiotherapist must identify incorrect movement patterns and needs to develop creative strategies, such as, for example, the sit-to-stand-exercise performed along a wall to prevent a lateral shift. Thus, patients may benefit from contact to a physiotherapist early in the course of OA, as at-home and multimodal management strategies can be optimized, even if routine physiotherapy visits are not needed yet.

As OA is a chronic and progressive disease, the patient needs lifelong support (e.g., by an exercise program). Therefore, in a holistic approach, the physiotherapist plays an important role in the periodical reassessment of the patient’s condition changing over time. Thus, they ensure the adaption of the multidisciplinary treatment of OA, when necessary.

## 4. Efficacy of Modalities Applicable by Physiotherapists

The term “therapeutic modalities” refers to the use of thermal, mechanical, electromagnetic, light or other energies with the goal to achieve specific therapeutic effects [118]. Numerous therapeutic modalities such as laser, cryo- and thermotherapy, extracorporeal shockwave, magnetic field, radiofrequency and electrotherapy have been recommended as adjunct measures to therapeutic exercises, manual therapy and patient owner education in dogs with osteoarthritis [12,119,120]. The majority of modalities are technically complex and need to be performed under direct supervision or personally by the physiotherapist. Simple cryo- and thermotherapeutic measures, which can be executed by the patient owner and magnetic field therapy, were beyond the scope of this review and are discussed elsewhere.

### 4.1. Low Level Laser Therapy (LLLT) in Osteoarthritis

The term LLLT refers to a painless non-invasive therapeutic modality, operating with near-infrared or infrared-light. In North America, LLLT is extremely popular for the therapy of canine OA and OA-related musculoskeletal disorders in veterinary practice [121,122]. The annual economic impact of laser therapy for treatment of a single OA joint in dogs has been estimated to be approximately $6.2 million per year for one single Midwestern U.S. state [121]. This is remarkable, as therapeutic efficacy of LLLT for OA has not yet been clearly determined yet.

The therapeutic effects of LLLT for patients with OA are not completely understood, but analgesic and anti-inflammatory effects have been demonstrated in experimental settings. In a rat model of peripheral inflammation, LLLT induced an analgesic effect via enhancement of peripheral endogenous opioid production in inflamed tissues [123]. Furthermore, the analgesic effect is thought to be mediated by direct inhibition of peripheral nerves [124]. Based on a systematic literature review, LLLT slows conduction velocity, reduces amplitude of compound action potentials and suppresses electrically and noxiously evoked action potentials in mammalian nerves [124]. In several rat models of artificially induced OA [31,125], laser therapy alone or combined with exercise promoted an anti-inflammatory effect due to improved microcirculation resulting in “wash out” of proinflammatory substances [125,126]. Significant reduction of proinflammatory substances such as PGE2, IL-1β and TNF-α, has so far been only observed with tendon injuries and muscle inflammation [127]. However, this anti-inflammatory effect has not been demonstrated in OA models yet.

In veterinary medicine, evidence for LLLT’s effectiveness in OA patients is still limited. One randomized blinded placebo-controlled trial [42] reported improved lameness scores and pain in dogs with naturally occurring elbow OA. In a retrospective study without control group, dogs with various affected OA joints responded positively to LLLT based on Canine Brief Pain Inventory (CBPI) and Visual Analogue Scale (VAS) scores. This positive effect was already observed after the first therapy session, but further improvement was observed with subsequent sessions. Analgesic medications could be reduced subsequently (see Table 1) [41].

In human medicine, the evidence base for therapeutic efficacy of LLLT for OA is better, but results are conflicting. Based on a recent Cochrane review evaluating seven controlled clinical LLLT trials in people with OA, evidence for LLLT’s potential therapeutic effect is inconclusive and insufficient considering the contradictory results of these studies [128]. However, laser therapy did not cause any side effects in neither human nor veterinary OA patients.

Application modes differ in LLLT studies for human and veterinary OA. Due to differing laser mode (continuous vs. pulsed), wavelengths, dosage and site of application (over nerves versus joints), comparison between studies and extrapolation is difficult. In particular, wavelength is an important parameter with regard to tissue penetration. Wavelengths between 650 and 1350 nm have the lowest absorption by the main chromophores (water, melanin, hemoglobin) and thus the highest penetration depth into tissues [129]. Nevertheless, there is still a lack of knowledge on the optimal joint-specific wavelength for OA treatment.

### 4.2. Therapeutic Ultrasound

Therapeutic ultrasound (TU) is a therapeutic modality employing acoustic sound waves in a non-audible frequency range between 1–3 MHz [130]. Therapeutic ultrasound induces thermal (by friction) and non-thermal (micro massage) effects [131,132]. Traditionally, TU’s deep tissue warming effect has been utilized in dogs to treat OA induced muscle shortening and hypertonus [133]. Due to the short duration of its heating effect, TU is typically used immediately prior to or during stretching in dogs with OA [134].

Recent experimental studies on the underlying molecular and genetic mechanisms indicate that TU exhibits complex anti-inflammatory effects mediated by gene induction, immunosuppressor cell promotion, and enhancement of exosome biogenesis and docking, that may prove to be of therapeutic benefit in aseptic inflammatory diseases such as OA [135]. Promising cellular effects were observed in human osteoarthritic articular knee cartilage compared to untreated controls: increased expression of type II collagen and proteoglycan, decreased matrix metalloproteinase-13 expression and increased chondrocyte proliferation [136,137]. Considering these effects, patients may also benefit from direct treatment of osteoarthritic joints.

In people, articular TU application has been shown to be a safe modality to reduce pain and to improve function in patients with knee OA, but this effect was only evaluated in a short time period between two weeks and one year [136]. A recent canine study indicated that TU may also be beneficial in dogs with OA. In an experimental canine stifle OA model (injections of sodium monoiodoacetate in the left knee, right joint served as control), effected joints were treated nine times with TU (3 MHz frequency, power density 1.20 W/cm^2^, pulsed wave duty cycle 1:2) [138]. Subsequently, improved synovial fluid viscosity, joint range of motion and muscle mass and increased loading on the affected limb were observed.

Evidence on therapeutic efficacy of selected TU mode (continuous versus pulsed), optimization of frequency, power density and dosing intervals for OA is still scarce. Findings on a recent systematic review and metanalysis indicated that pulsed TU may be more efficacious than continuous TU for pain control and improved function in people with knee OA [139]. Further controlled comparative studies are needed.

### 4.3. Extracorporeal Shock Wave Therapy

Extracorporeal shock wave therapy (ESWT) is a therapeutic modality employed to treat various musculoskeletal diseases including tendinous disorders, fasciitis, non-union of long bone fractures and avascular necrosis of the femoral head and osteoarthritis [140]. Shockwaves are highly energetic acoustic waves with very high-pressure amplitude and ultrashort duration. In veterinary medicine, focused and radial shockwave technologies have been employed for the treatment of OA [37,38,39,40]. Focused shockwaves are generated by electrohydraulic, electromagnetic and piezoelectric devices based on shock wave lithotripter technology and unfold their highest energy density deep in the tissues [141,142]. Radial shockwaves, on the other hand, are generated by ballistic devices and have a more superficial unfocussed effect on tissues with a decrease in energy in proportion to the square of the distance from the surface [39,141].

Shockwaves are thought to provide mechanical stimuli resulting in biologic tissue effects. ESWT induced cellular alterations result from conversion of a mechanical signal into biochemical or molecular biologic signals (mechanotransduction) [143]. The mechanisms of action of ESWT have not been entirely understood yet, but the beneficial effect of shockwaves for OA therapy appears to be based on chrondroprotective and analgesic effects [140].

In early knee arthritis in rats, ESWT significantly reduced cartilage surface damage and proteoglycan loss, enhanced subchondral bone repair upon micro-CT evaluation and promoted cartilage proliferation [144]. Based on an experimental study in rabbits, ESWT lessened the progression of knee OA by reduction of nitric oxide level and chondrocyte apoptosis [32]. In an OA knee model in rats, ESWT showed a positive effect decreasing metalloproteinase and increasing type II collagen synthesis and anabolism, as well as blood flow to the subchondral bone in a number of treatment related manners [145]. Several experimental studies also showed ESWT induced neovascularization and upregulation of angiogenic and osteogenic growth factors (endothelial nitric oxide synthase, vascular endothelial growth factor, proliferating cell nuclear antigen, and human bone morphogenetic protein 2) in bone healing and tendinopathy models [146,147].

The pathomechanism of ESWT’s analgesic effect has not been completely elucidated yet. Hyperstimulation of nociceptors and the interruption of nerve impulses have been postulated to trigger an analgesic effect in patients with chronic pain [143]. Several studies point towards degeneration of nerve endings inhibiting the upward transmission of pain via sensory nerve fibers as ESWT’s analgesic pathomechanism [148,149]. Non-focused ESWT of the palmar digital nerves in horses decreased sensory nerve conduction velocities [150]. In this study, disruption of the myelin sheath with no evidence of damage to Schwann cell bodies or axons of the treated nerves was observed upon transmission electron microscopy. Thus, simultaneous ESWT of nerves adjacent to treated osteoarthritic joints may also contribute to ESWT induced analgesia [150]. Shockwaves may also ameliorate pain in osteoarthritic patients, as ESWT reduces the expression of calcitonin gene-related peptide in dorsal root ganglia, which play a role with sensation of joint pain [149,151]. Some authors have theorized that the analgesic effect may be associated with ESWT induced enhancement of the microcirculation [152].

In prospective clinical studies, ESWT improved lameness upon objective gait analysis in dogs with elbow and hip OA, but not in dogs with stifle OA compared to the control groups (see Table 2) [37,38,39,40]. Except for Souza et al.’s study on radial ESWT for hip osteoarthritis, these canine studies have pilot character due to lower patient numbers. Although focused ESWT improved PVF and VI in dogs with osteoarthritic elbows significantly compared to the control group, study duration was limited to 28 days and long-term therapeutic effects are still unknown [38]. Radial ESWT has been shown to benefit dogs with hip OA in 2 studies up to 3 and, respectively, 6 months [39,40]. Nevertheless, evidence of Mueller et al.’s study is considered limited due to the low case number and the non-randomized and non-blinded study design [39,153]. There is an obvious lack of studies investigating long-term effects, optimal dosing intervals, intensity and frequency for radial and focused ESWT for dogs with OA.

Although studies investigating clinical benefits of ESWT in osteoarthritic dogs are limited, numerous studies exploring therapeutic efficacy of ESWT in people with OA have been performed and systematic reviews and metanalyses are encouraging. A recent systematic review and metanalysis focused on the efficacy of ESWT for all types of human OA [154]. Based on this review, ESWT was superior in both pain reduction and functional improvement compared with placebo, corticosteroid, hyaluronic acid, medication and ultrasound [154]. In people with knee OA, ESWT was considered effective in reducing pain and improving functionality based on a recent systematic review and metanalysis. Nevertheless, further studies were requested by authors, as well as commentators to investigate ESWT’s long-term effects and to determine optimal dosing intervals, intensity and frequency for treatment of knee OA [155].

In general, ESWT is considered a safe and non-invasive modality with minimal side effects. However, in a randomized controlled trial on people with low to moderate knee OA, low-dose ESWT for 4 weeks resulted in T2 changes indicative for cartilage degradation upon MRI. As T2 changes did not differ significantly between placebo and ESWT, the authors concluded that ESWT is still an effective and safe modality for pain reduction and functional improvement in this particular patient group [155].

In canine practice, focused ESWT has not been as well tolerated as radial ESWT and in the past required sedation in the majority of dogs. Meanwhile, novel trode technologies and gradual increase of shockwave intensity and frequency throughout focused ESWT sessions may allow clinicians to sidestep the need for sedation in dogs (personal experience of authors).

### 4.4. Electrotherapy

#### 4.4.1. Electrotherapy in Canine Osteoarthritis

The field of electrotherapy is very multifaceted and includes various types of applications differing between direct and alternating current. The latter is further divided into low (0–1000 Hz, e.g., transcutaneous electrical nerve stimulation, neuromuscular electrical stimulation), middle (1–100 kHz, e.g., interferential current) and high frequency (>100 kHz, e.g., microwave) applications. Patients with OA may benefit from several electrotherapeutic effects. An analgesic effect has been induced via direct stimulation of peripheral nerves [156] but has also been mediated by release of endorphins [157]. Electrotherapy may counteract secondary OA-induced periarticular muscular alterations: enhancing muscular activation, normalizing muscle tone and increasing metabolism [157] and blood flow [108]. Frequently, human OA guidelines erroneously equate the term electrotherapy with the technique of neuromuscular electrical stimulation (NMES). This is an inadequate simplification. As study results on NMES efficacy for OA are conflicting, these guidelines may rate electrotherapy as “not appropriate” or may not mention it at all subsequent to this simplification [19,22]. However, other electrotherapeutic interventions such as interferential current have been considered promising for OA pain relief [158], while evidence for therapeutic efficacy of microwave therapy for OA is scarce in human and veterinary medicine and currently no recommendation can be made. Therefore, the three most common electrotherapy techniques applied in veterinary medicine are discussed in the following section.

#### 4.4.2. Interferential Current

Interferential current (IC) is characterized by the diagonal crossing of two medium frequency alternating current loops (about 4000 Hz) with a difference in frequency (1–100 Hz). At the intersection of the two circuits, continuous phase shifts occur due to the frequency difference and initiate a low- frequency current (0–250 Hz). This low- frequency therapy allows for deep tissue penetration and can be applied without skin irritation to joints [159].

The evidence in canine OA includes two studies: a randomized placebo controlled cross-over clinical trial investigated the effect of IC on ground reaction force in dogs with hip osteoarthritis (100 Hz, 250 µs pulse duration, phase duration of 125 µs) [36]. The IC-treated dogs showed a significant increase in peak vertical force, but the sample size was small (*n* = 9) and there was no control group. An observational study in five dogs with musculoskeletal pain reported a good outcome regarding pain and observed increased mobility [159]. The best analgesic effect was reported for an intensity of 6 mA and a frequency of 80–100 Hz. Furthermore, a series of mild local muscle contractions was seen during the treatment with local hyperaemia, and the dogs showed a state of comfort.

A recent systematic review indicated that IC is superior for pain management of osteoarthritic knees in people, when compared with five other electrotherapeutic modalities [158]. Other IC-induced positive therapeutic effects for OA include oedema reduction and reduction of muscle spasms [159].

#### 4.4.3. Neuromuscular Electrical Stimulation

Muscle strengthening is an important physiotherapeutic goal, as OA is associated with muscle atrophy and weakness. Neuromuscular electrical stimulation (NMES) is an electrotherapeutic intervention that can be utilized as an alternative to exercise for muscle strengthening, when patients are too painful or weak or have comorbidities prohibiting exercise [160]. Neuromuscular electrical stimulation has been recommended as modality for osteoarthritic dogs, although there is currently only extrapolated evidence supporting NMES in this patient group [156]. In a canine experimental model with severed cranial cruciate ligament, dogs treated with NMES showed improved lameness scores, larger thigh circumference, less crepitation and fewer radiographic OA changes compared to the control group [33]. Similar positive effects have been observed in people treated with NMES for knee OA, showing increased thickness and fascicle length in the vastus lateralis muscle, improved joint extension and stiffness and improved comfort and function compared after NMES therapy [161]. In a randomized controlled trial on 41 people with knee OA, home-based NMES showed significant improvement in function and muscle mass compared to the conventional exercise group and similar improvements compared to home-based resistance training [162]. However, a systematic comparative review did not confirm a significant NMES-induced pain reduction for human knee OA [158]. In people with knee OA, adherence to NMES based therapy has not been superior or inferior to voluntary exercise programmes with patient education [163]. Currently, NMES frequencies of 25–50 Hz with 150 and 250 ms pulse duration are recommended for muscular strengthening in people, while dogs often tolerate lower frequencies (<10 Hz) better [156].

#### 4.4.4. Transcutaneous Electrical Nerve Stimulation

Transcutaneous electrical nerve stimulation (TENS) is a low frequency electrotherapeutic modality with mono- or (mostly) biphasic square-wave pulses (alternating current). The main mode of action is activation of a complex neuronal network by activating descending inhibitory systems in the central nervous system to reduce pain. This modality can be applied both locally or at the corresponding spinal segment. Conventional TENS (=h-TENS, 50–150 Hz, 2–50 µs pulse duration, low intensity, effect via Gate-Control-mechanism) and the so-called acupuncture-like TENS (=l-TENS, 1–10 Hz, 100–400 µs pulse duration, high-intensity, effect via distribution of endogenous endorphins) are most frequently used [156]. Only a few studies investigate efficacy of TENS for OA therapy in veterinary medicine. In several murine studies, TENS produced an analgesic effect in experimentally induced OA [164,165,166,167,168]. In a pilot study on dogs, the effect of h-TENS (70 Hz) was investigated after extraarticular stabilization of cranial cruciate ligament deficient osteoarthritic stifle joints. In this study, ground reaction forces improved significantly in the affected limbs starting immediately after h-TENS application. Although this significant effect persisted until 210 min after application, it was highest immediately after application and was not observed anymore upon a four day recheck [169]. In a non-blinded prospective randomized clinical trial on 29 overweight dogs with various osteoarthritic joints enrolled in a standardized weight loss protocol, dogs undergoing a more intense PT program with biweekly TENS had a significantly more even peak vertical force symmetry between affected and non-affected limbs compared to dogs undergoing a regular outpatient PT program from day 60 until the end of the study [170]. However, there was no significant difference between pain scores and vertical impulse symmetry between groups. It remained unclear whether the positive effects observed were associated with TENS application or the more intense PT care with add-on supervision and in-house PT sessions. Empirically, TENS with high intensity and longer treatment duration (2–3 times per week, for 5–6 weeks, each session 30 min) is recommended for dogs with chronic conditions such as OA [59].

In human medicine, systematic reviews on efficacy of TENS for OA are contradictory. Whereas one review “could not confirm that transcutaneous electrostimulation is effective for pain relief in osteoarthritic knee joints” [171], other authors concluded that TENS is effective [172]. This discrepancy is probably associated with the current suboptimal study quality (small sample size, poor methodological quality, inadequate randomization and blinding) [168,173]. Nevertheless, the National Institute for Health and Care Excellence in the UK currently recommends TENS for OA pain in humans (last update in 2020).

#### 4.4.5. Capacitive-Resistive Electric Transfer (CRET)

The term capacitive-resistive electric transfer (CRET) refers to a non-invasive electro-thermal therapeutic modality, where electric currents are applied within the radiofrequency range of 400–450 kHz [174]. Capacitive-resistive electric transfer has been utilized in human patients with muscle, bone, ligament and tendon lesions with the goal to decrease pain [174,175,176,177]. This modality utilizes a generator of long-wave currents and electrodes with multifrequency sequential systems of emission to transfer energy to tissues in capacitive and resistive modes [175]. Superficial tissues are targeted in capacitive mode, while deeper tissues such as tendons, bone and cartilage are affected in resistive mode. CRET has been shown to elicit thermal effects subsequent to the electrical resistance of tissues but will not cause overheating due to heat dissemination by circulating blood [174,178,179,180]. Capacitive-resistive electric transfer improves blood circulation and subsequently allows for enhanced evacuation of inflammatory catabolites and tissue relaxation [180].

Osteoarthritic patients treated with CRET may benefit from this deep thermal effect (see therapeutic ultrasound), but also from a direct cellular effect associated with subthermal CRET currents. Subthermal CRET at 448 kHz promotes proliferation of human mesenchymal stem cells in injured tissues based on in vitro studies [174].

Literature on CRET application for OA is currently limited, but superior pain control and/or functional improvements were observed in two randomized prospective double-blinded studies on people with mild to moderate knee osteoarthritis [175,181]. In one study, six CRET sessions of 20 min duration improved strength, function and pain control significantly directly after the six treatments and after 1 and 3 months compared to the sham treated control group [175].

Further studies are needed to confirm efficacy for different joints, more severe OA and different species. Optimal therapeutic CRET parameters for OA therapy still have to be determined.

## 5. Discussion

Osteoarthritis is a global canine health problem. In human medicine, general practitioners are considered to be the main care providers for OA patients [182]. Thus, providing evidence-based guidelines for practitioners is imperative to improve quality of care of OA management [182]. This statement appears equally applicable to canine OA management. The authors intended to weigh the impact of different physiotherapeutic measures on dogs with OA utilizing the concept of the “Physiotherapy Pyramid”. Similar concepts have been established for human OA therapy [27]. In human medicine, such guidelines ideally take cost effectiveness, short and long-term clinical effectiveness, as well as relative therapeutic efficacy and time requirements for therapeutic measures into consideration.

In veterinary medicine, evidence-based information on efficacy of physiotherapeutic measures is still scarce but constantly evolving. Subsequently, the current “Physiotherapy Pyramid” concept proposed in this review is based on common sense, evidence based veterinary information and information extrapolated from the human OA literature. However, the jury is definitely still out with regard to relative therapeutic efficacy of the different available therapeutic measures. Adaptations of the pyramid are needed depending on the evidence rising from future research.

Human OA is a disease with chronic non-curable character associated with very high healthcare cost. Considering this enormous economic burden, cost-effectiveness of physiotherapeutic measures in OA patients has become an important factor that is routinely evaluated in human medicine [183].

In veterinary medicine, the economic impact of OA has been calculated for an equine population [184] but has not been established for dogs yet [121], except for estimations by the veterinary health industry and insurances. Studies on the cost for physiotherapeutic measures spent for osteoarthritic dogs are missing, except for LLLT in a Midwestern state in the United States [121]. To the authors’ knowledge, there is a complete lack of studies evaluating cost efficacy of PT measures for osteoarthritis in dogs.

Studies on acute and short term clinical effectiveness of PT measures currently predominate the veterinary literature [42,43,107,169] and often have only an observational character [41,159]. Base of evidence tends to be low due to lack of control groups and/or low case numbers [36,37,38,39]. Rarely therapeutic efficacy has been investigated for 6 months or longer [39]. To the authors’ knowledge, clinical effectiveness for OA therapy in dogs has not been evaluated yet for passive range of motion exercises, massage therapy, therapeutic ultrasound, NMES and CRET. A positive effect of LLLT in osteoarthritic dogs has been shown in a randomized blinded placebo-controlled clinical trial of 6 weeks duration in dogs with elbow OA clinical study [42]. Only one further retrospective study investigated LLLT-induced positive therapeutic effects in osteoarthritic dogs for a duration of only two weeks following a 6-week course of LLLT therapy [41]. Although results of these two LLLT studies are promising, further controlled studies are needed to investigate the long-term effect of LLLT in osteoarthritic dogs and to determine optimal LLLT parameters and dosage intervals for canine OA depending on joint and degree of OA. As studies evaluating clinical efficacy of IC in osteoarthritic dogs still have pilot character [36,159], further studies with larger sample size and control groups are needed for definitive recommendations. Similarly, most ESWT studies on osteoarthritic dogs are limited due to low case numbers [37,38,39] except for one study on hip OA [40]. Superior efficacy of focused versus radial ESWT for OA in dogs has not been determined yet. Adherence (compliance) to therapeutic recommendations is of utter importance for therapeutic success in patients with OA. Factors affecting adherence of patient owners to PT plans for their dogs have not been established yet, but patient and owner comfort throughout therapeutic interventions, financial considerations and time requirements are expected to play an important role. Minimization of time required for treatments to be performed by the owner and for visits with the canine physiotherapist may positively affect long-term adherence of owners with dogs suffering from chronic diseases such as OA. New solutions, including blended PT interventions partially replacing face to face PT sessions by apps and instructions from websites, may have the potential to decrease cost and to increase long-term adherence in canine OA patients [183]. In conclusion, there is a substantial need for further studies on optimal dosing regimen, treatment duration, relative therapeutic and cost efficacy for physiotherapeutic measures, compliance and long-term success of PT measures to optimize physiotherapeutic guidelines and recommendations for dogs with OA.

## Figures and Tables

**Table 1:** Treatment parameters for OA in affected joints.

Study	Mode	Wavelength	Intensity	Frequency	Treatment Duration
Looney et al. [42]	continuous	650 nm and 980 nm	10–20 J/cm^2^	2/week	6 weeks
Barale et al. [41]	continuous and pulsed	808 nm	5 J/cm^2^ (affected joint) 4.2 J/cm^2^ (associated skeletal muscle)	1/week	6 weeks

**Table 2:** Extracorporeal shockwave therapy (ESWT) for canine osteoarthritis (Abbreviations: PVF: peak vertical force; VI: vertical impulse).

Authors	Dogs Number	OA	ESWT Therapy Technical Details	Clinical Follow-Up	Effect
Dahlberg et al. (2005) [2,37]	ESWT = 7 Control = 5	Stifle	800 pulses, focal pressure depth: 20 mm; 700 pulses: focal depth: 5 mm 4 Hz, energy flux density: 0.14 mJ/mm^2^, 3 times every 3 weeks	98 days	No significant improvement for PVF, VI and owner questionnaires
Mueller et al. (2007) [39]	ESWT = 18 Control = 6	Hip	Radial, 2000 pulses, 15 Hz, 2 bars, 3 times every 7 days	6 months	Improved PVF, VI
Millis et al. (2011) [38]	ESWT = 8 Control = 7	Elbow	Focused, 500 pulses, 5 mm probe, energy flux density: 0.13 mJ/mm^2^, twice every 2 weeks	28 days	Improved PVF, lameness scores
Souza et al. [40]	ESWT = 30 Control = 30	Hip	Radial, 2000 pulses, 10 Hz, 2–3.4 bars, 3 times every 7 days	3 months	Improved PVF and VI, VAS and activity

## Data Availability

Not applicable.
